# Fine-Grained Classification of Pressure Ulcers and Incontinence-Associated Dermatitis Using Multimodal Deep Learning: Algorithm Development and Validation Study

**DOI:** 10.2196/67356

**Published:** 2025-05-01

**Authors:** Alexander Brehmer, Constantin Seibold, Jan Egger, Khalid Majjouti, Michaela Tapp-Herrenbrück, Hannah Pinnekamp, Vanessa Priester, Michael Aleithe, Uli Fischer, Bernadette Hosters, Jens Kleesiek

**Affiliations:** 1 Institute for Artificial Intelligence in Medicine, Essen University Hospital, Girardetstr. 2, Essen, 45131, Germany, 0201 72377829; 2 Center for Virtual and Extended Reality in Medicine, University Medicine Essen, Essen, Germany; 3 Faculty of Computer Science, University of Duisburg-Essen, Essen, Germany; 4 Department of Nursing Development and Nursing Research, University Hospital Essen, Essen, Germany; 5 Department of Clinical Nursing Research and Quality Management, Hospital of the Ludwig Maximilian University, Munich, Germany; 6 Sciendis GmbH, Leipzig, Germany

**Keywords:** computer vision, image classification, wound classification, deep learning, pressure ulcer, incontinence-associated dermatitis, multi modal data, synthetic image generation

## Abstract

**Background:**

Pressure ulcers (PUs) and incontinence-associated dermatitis (IAD) are prevalent conditions in clinical settings, posing significant challenges due to their similar presentations but differing treatment needs. Accurate differentiation between PUs and IAD is essential for appropriate patient care, yet it remains a burden for nursing staff and wound care experts.

**Objective:**

This study aims to develop and introduce a robust multimodal deep learning framework for the classification of PUs and IAD, along with the fine-grained categorization of their respective wound severities, to enhance diagnostic accuracy and support clinical decision-making.

**Methods:**

We collected and annotated a dataset of 1555 wound images, achieving consensus among 4 wound experts. Our framework integrates wound images with categorical patient data to improve classification performance. We evaluated 4 models—2 convolutional neural networks and 2 transformer-based architectures—each with approximately 25 million parameters. Various data preprocessing strategies, augmentation techniques, training methods (including multimodal data integration, synthetic data generation, and sampling), and postprocessing approaches (including ensembling and test-time augmentation) were systematically tested to optimize model performance.

**Results:**

The transformer-based TinyViT model achieved the highest performance in binary classification of PU and IAD, with an F1-score (harmonic mean of precision and recall) of 93.23%, outperforming wound care experts and nursing staff on the test dataset. In fine-grained classification of wound categories, the TinyViT model also performed best for PU categories with an F1-score of 75.43%, while ConvNeXtV2 showed superior performance in IAD category classification with an F1-score of 53.20%. Incorporating multimodal data improved performance in binary classification but had less impact on fine-grained categorization. Augmentation strategies and training techniques significantly influenced model performance, with ensembling enhancing accuracy across all tasks.

**Conclusions:**

Our multimodal deep learning framework effectively differentiates between PUs and IAD, achieving high accuracy and outperforming human wound care experts. By integrating wound images with categorical patient data, the model enhances diagnostic precision, offering a valuable decision-support tool for health care professionals. This advancement has the potential to reduce diagnostic uncertainty, optimize treatment pathways, and alleviate the burden on medical staff, leading to faster interventions and improved patient outcomes. The framework’s strong performance suggests practical applications in clinical settings, such as integration into hospital electronic health record systems or mobile applications for bedside diagnostics. Future work should focus on validating real-world implementation, expanding dataset diversity, and refining fine-grained classification capabilities to further enhance clinical utility.

## Introduction

### Background

Pressure ulcers (PUs) and incontinence-associated dermatitis (IAD) are significant challenges in clinical settings due to their prevalence and impact on patient health and well-being. The global prevalence of PUs is estimated to be 12.8% [1], while studies have estimated the IAD prevalence to be between 5.6% and 50% [2]. These wounds not only cause physical discomfort but also pose risks of infection and prolonged hospital stays, increasing health care costs and diminishing the quality of life for affected individuals.

Accurately distinguishing between PUs and IAD poses a considerable challenge for health care providers and wound care experts. Both conditions share similar presentations, yet their underlying causes and optimal treatment approaches differ vastly. This ambiguity not only complicates diagnosis but also delays appropriate interventions, potentially exacerbating patient discomfort and prolonging healing times [3].

To address this challenge, the KIADEKU project [4] was initiated to develop an innovative artificial intelligence (AI) system capable of distinguishing between PUs and IAD using wound image data and key patient information.

### Goal of This Study

The goal of this study is to advance wound care by developing a robust multimodal deep learning framework for the fine-grained classification of PUs and IAD. By integrating wound images with categorical patient data, we aim to enhance diagnostic accuracy in distinguishing between these conditions and in categorizing their respective wound severities. We conduct extensive benchmarking of state-of-the-art convolutional and transformer-based models, emphasizing optimal performance while ensuring computational efficiency for practical deployment in clinical settings. The optimized model addresses the challenging task of accurately classifying PU and IAD wounds, providing valuable insights and tools to support clinical decision-making and guide future research in wound classification.

### Related Work

Deep learning has significantly advanced wound classification, including PUs and other wound types. Various studies have explored different deep learning architectures and techniques to improve diagnostic accuracy and efficiency. Table 1 summarizes key contributions in this domain.

While previous studies have demonstrated the effectiveness of deep learning for wound classification, they predominantly rely on image data alone. However, accurate wound diagnosis often depends on both visual appearance and key clinical factors, such as wound location, patient mobility, and incontinence severity. To our knowledge, no existing study rigorously integrates multimodal data fusion, combining wound images with categorical patient information. Our approach leverages this additional patient context, allowing the model to capture clinically relevant patterns that purely image-based models may overlook, thereby significantly improving diagnostic precision and decision support. Furthermore, our approach involves extensive benchmarking of state-of-the-art convolutional and transformer-based models, as well as various training techniques, augmentations, and postprocessing methods to enhance performance. This comprehensive evaluation sets our method apart in both scope and effectiveness, contributing to a novel multimodal framework for fine-grained wound classification that can support clinical decision-making and guide future research in this domain.

**Table 1.** Summary of related work in wound classification and pressure ulcer classification.

Authors	Method	Key contributions
Pressure ulcer classification		
Aldughayfiq et al [5][Bibr R5]	YOLOv5-based classification	Classified pressure ulcers into 4 stages and non-pressure ulcer categories with real-time detection capabilities.
Chang et al [Bibr R6][6]	Superpixel segmentation	Used superpixel techniques for automatic pressure ulcer diagnosis, enhancing wound segmentation and classification accuracy.
Seo et al [Bibr R7][7]	CNN^a^-based classification	Developed a deep learning model to visually classify pressure injury stages, aiding nurses in diagnostic accuracy.
García-Zapirain et al [Bibr R8][8]	3D CNNs	Explored 3D CNNs for classifying pressure ulcer tissues, capturing spatial features for precise tissue type classification.
Liu et al [Bibr R9][9]	CNN-based assessment system	Introduced a system to aid in pressure ulcer diagnosis and clinical decision-making, enhancing speed and accuracy.
Lau et al [Bibr R10][10]	AI^b^-enabled smartphone app	Developed an app for real-time pressure injury assessment using advanced AI algorithms.
Kim et al [Bibr R11][11]	Deep learning model for staging	Assessed a deep-learning model’s clinical utility for pressure injury staging, enhancing decision-making in wound care.
Swerdlow et al [Bibr R12][12]	Mask R-CNN	Proposed simultaneous segmentation and classification of pressure injury images, improving diagnostic efficiency.
Zahia et al [Bibr R13][13]	CNNs for classification	Focused on classification and segmentation of pressure injury tissues, identifying different tissue types accurately.
Pandey et al [14]	Thermal imaging classification	Developed and validated a deep learning-based thermal imaging framework to automatically stage pressure ulcers
Wound classification		
Huang et al [Bibr R14][15]	CNN-based tool	Developed a tool for automatic classification of various wound types, supporting accurate diagnoses.
Oura et al [Bibr R15][16]	Deep learning in forensic analysis	Applied deep learning for gunshot wound interpretation, demonstrating versatility in wound classification contexts.
Rostami et al [17]	Ensemble CNN classifier	Explored multiclass wound image classification using ensemble methods to enhance accuracy.
Patel et al [Bibr R17][18]	Integrated image and location analysis	Incorporated visual and locational data for wound classification, highlighting multimodal data integration’s importance.
Liu et al [Bibr R18][19]	EfficientNet models	Applied EfficientNet to classify diabetic foot ulcer ischemia and infection, handling complex wound classification tasks.
Lee et al [Bibr R19][20]	Ultrasound imaging with deep learning	Developed a model for burn depth classification using ultrasound images for non-invasive assessment.
Afza et al [Bibr R20][21]	Hybrid deep features selection	Investigated skin lesion classification using deep features and extreme learning machines, enhancing medical image analysis.
Cheng et al [22]	ConvNext Tiny, Gun Shot Classification	Pioneers the application of deep learning in forensic pathology by demonstrating that AI can reliably differentiate between entrance and exit gunshot wounds using digital color images.
Odame et al [23]	CLAHE-enhanced images, DWT, FixCaps	Developed a multi‐wound classification framework that integrates image enhancement (using CLAHE and DWT) with deep learning

a CNN: convolutional neural network.

b AI: artificial intelligence.

## Methods

### Dataset

In this study, we use a new wound dataset collected and annotated over a 2-year period as part of the KIADEKU project. The data originate from the project partners Ludwig Maximilian University University Hospital and Essen University Hospital and were annotated by 4 wound experts with extensive clinical experience in wound management using the Label Studio Software [24]. Considering the difficulty of the task, we enforced a strong ground truth by having all images annotated by 3 wound experts and only used images where 2 wound experts reached consensus in their annotations.

**Figure 1.**
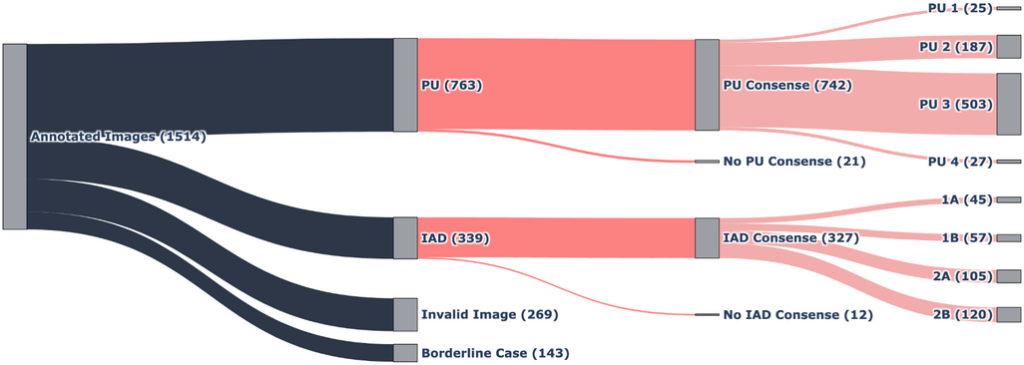
Dataset composition with distribution of wound types and categories. PU: pressure ulcer; IAD: incontinence-associated dermatitis.

The annotators categorized each image as either IAD, PU, invalid, or borderline case (both wounds present) and assessed the categorization of each wound type. For PU classification, we followed the *International Classification of Diseases*-10 standard [25], which defines 4 degrees (1-4) of PU wounds. Similarly, for IAD classification, we used the Ghent Global IAD Categorization Tool (GLOBIAD) [26], which categorizes IAD wounds into 4 distinct categories: 1A, 1B, 2A, and 2B. Figure 1 shows an exemplary annotation interface.

**Figure 2.**
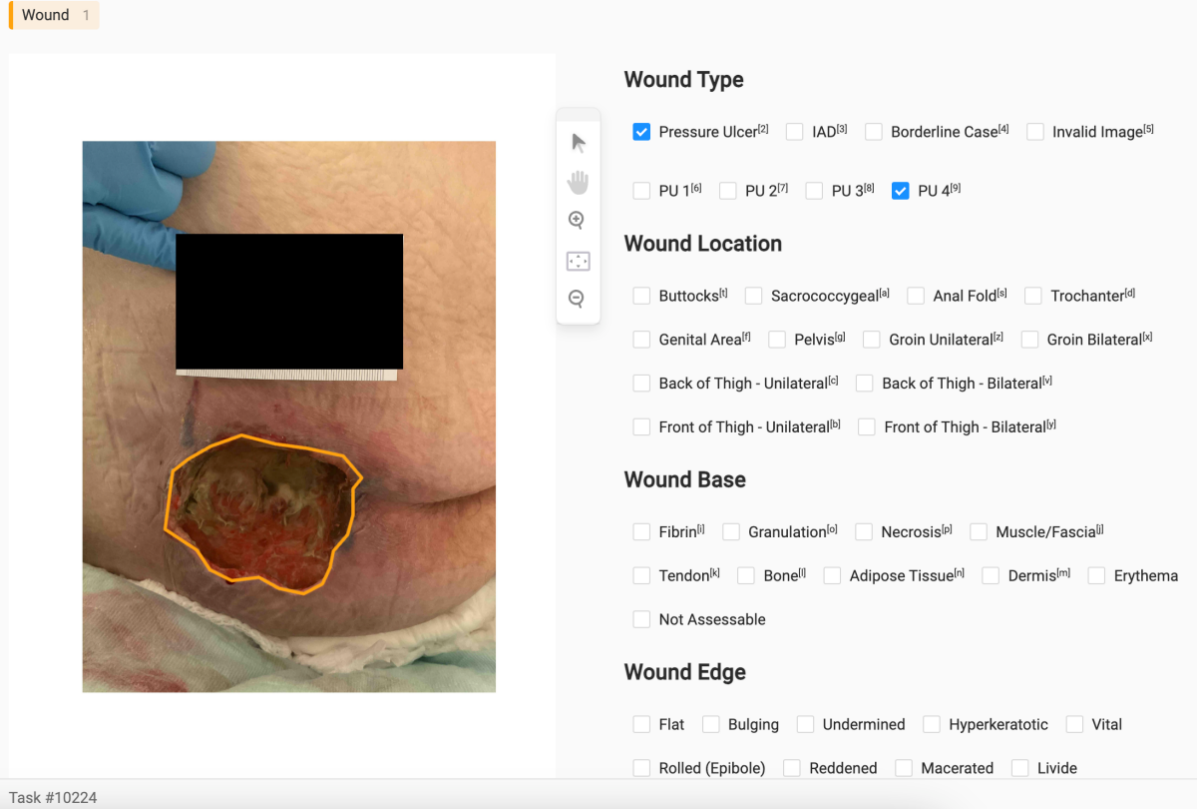
Exemplary annotation process in Label Studio. PU: pressure ulcer; IAD: incontinence-associated dermatitis.

Employing the described annotation protocol, a dataset of 1555 images was annotated, from which 1514 images received consensus validation among the annotators. Analysis of the data revealed a generally balanced distribution between the 2 principal wound types under study, PUs and IAD, as depicted in Figure 2. The dataset comprised 763 images of PU and 339 images of IAD.

Of the 763 images categorized as PUs, consensus was achieved for 742 images regarding their specific PU category. The distribution of these categories, as illustrated in Figure 2, reveals a significant class imbalance. Notably, categories 1 and 4 are markedly underrepresented, containing only 25 and 27 images, respectively, compared to 187 images in category 2 and 503 in category 3. This pronounced disparity in class sizes is a critical factor that must be considered when interpreting the training results.

Of the 339 images initially classified as IAD, a consensus on the specific IAD category was reached for 327 images. The class distribution within these categories, as depicted in Figure 2, is relatively balanced compared to the distribution observed in PU categories. Category 2B is the most represented, with 120 images, followed by category 2A with 105 images, 1B with 57 images, and 1A with 45 images. Although this distribution is less skewed than that observed in the PU categories, the smaller sample size overall remains a significant consideration for model training and validation.

### Methodology

**Figure 3.**
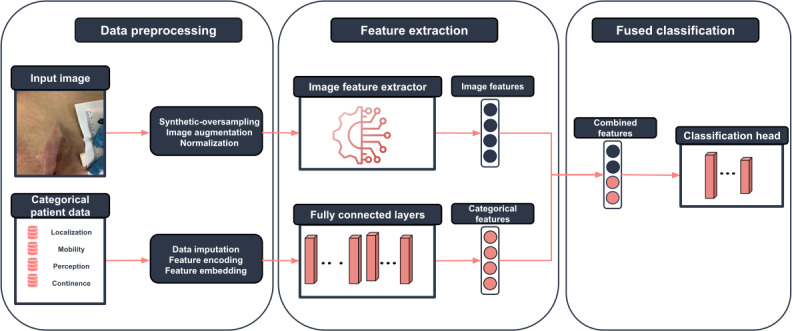
Multimodal architecture visualization illustrating the use of data preprocessing and feature extraction, before fusing the features for the final classification.

Our proposed classification framework is specifically designed to handle and classify both images and categorical data effectively, as shown in Figure 3.

Initially, the original images undergo several preprocessing steps. These steps include image augmentations and normalization to standardize the input data, alongside the generation of synthetic data points by fine-tuning a stable diffusion model and using these synthetic samples to oversample the minority classes across tasks. We then compare the performance of this approach with traditional oversampling techniques that rely on original data points. For categorical patient data (eg, wound location, mobility, perception ability, and continence status), missing values were addressed using mode imputation, where the most frequent value for each feature was assigned. In cases where missing values exceeded 20% of the dataset for a particular feature, the affected samples were excluded to prevent bias. Additionally, images with conflicting expert annotations (ie, cases where consensus was not reached) were removed to maintain ground truth integrity. After data preprocessing, we extract features from each modality. Image features are extracted using various feature extractors from the Timm [27] library, renowned for their robustness and efficiency; in parallel, categorical features are derived using a simple feed-forward neural network designed to capture the essential characteristics of the embedded categorical data.

In the final stage of our framework, we employ 3 distinct modality fusion techniques to integrate image and categorical features before classification. In the concatenation-based fusion, features from both modalities are directly concatenated to form a comprehensive feature set, which is then passed to a classification head. In the cross-attention-based fusion, categorical features are projected into the image feature space, and a multihead attention mechanism is applied to capture their interactions. In the gated fusion, a gating mechanism adaptively balances the contributions of both modalities, allowing the model to learn the optimal weighting before classification. Each approach ensures effective multimodal integration while leveraging different fusion strategies. The combined feature set is then fed into a final classification head, which is tasked with making the final prediction based on the integrated data.

This setup facilitates a systematic examination and evaluation of various data preprocessing strategies, training techniques, and postprocessing approaches, both independently and in combination. This rigorous methodology allows for a comprehensive comparison and assessment of their efficacy in various combinations across our designated tasks.

### Experimental Setup

**Figure 4.**
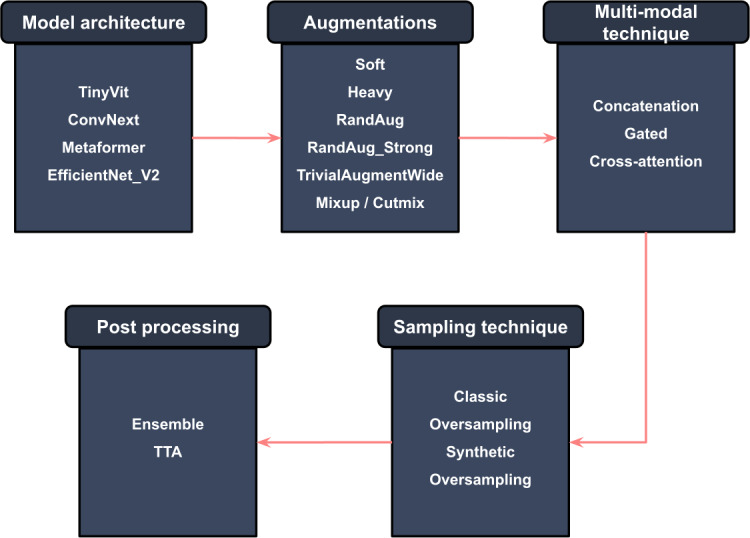
Visualization of experiment setup and benchmark flow. TTA: test-time augmentation.

To maintain a manageable number of experiments, we did not evaluate every possible combination. Instead, we benchmarked various components individually and sequentially integrated the optimal variations for subsequent tests. Specifically, we first identify the model architecture that achieves the highest average rank across our metrics and use this model as the basis for testing different augmentation techniques. The best performing augmentation variation, as determined by the average metric rank, is then used to assess different training techniques. Finally, the best combination of model, augmentation, and training technique is used to benchmark the most effective postprocessing strategy. For a visual representation of this benchmarking flow, refer to Figure 4.

### Training

The general training procedure involves setting the learning rate to 0.0001 and resizing the input images to 384x384 pixels. We use a batch size of 64, with the AdamW optimizer to manage weight decay, and the CrossEntropyLoss loss function for training. The learning rate is adjusted using a CosineAnnealingWarmRestarts scheduler, starting with a cycle length of 10 epochs and a minimum learning rate of 1e-6. Training is performed on an NVIDIA A100 graphics processing unit, with early stopping enabled and a patience of 15 epochs to prevent overfitting. The dataset is initially split into an 80:20 ratio for training and testing. The training set is further divided into 5 equal parts (folds) for cross-validation to enable robust model evaluation.

### Models

To evaluate and identify the best possible model for the binary classification task of IAD and PU, as well as the fine-grained wound category classification within these wound types, we selected 4 models with approximately 25 million parameters to ensure a fair comparison and fast inference speed. Using transfer learning, we employed pretrained models from the Timm library, which were originally trained on ImageNet [27]. Our selection includes 2 convolution-based models and 2 transformer-based models, chosen for their exceptional performance relative to their parameter count, as evidenced by Timm’s test results on the ImageNet benchmark. For the convolution-based models, we selected a pretrained ConvNeXtV2 model [28,29] and a pretrained EfficientNetV2 model [30,31]. These models are chosen for their state-of-the-art performance and efficiency, making them highly suitable for a wide range of computer vision tasks. The ConvNeXtV2 incorporates advanced architectural enhancements, while EfficientNetV2 uses a novel scaling approach for optimal accuracy and computational efficiency. For the transformer models, we included the MetaFormer [32] and TinyViT [33,34]. The MetaFormer is selected for its innovative design that enhances transformer capabilities, while TinyViT, a distilled vision transformer, is designed to retain high accuracy with fewer parameters and computational resources, making it suitable for resource-constrained environments.

### Augmentations

We evaluate 6 distinct augmentation techniques comprising 3 randomized methods and 2 custom-designed variants and the use of CutMix/MixUp [35]. Initially, we test the RandAugment [36] method using its PyTorch implementation with default settings. To explore more robust options, we employ an intensified version of RandAugment, increasing the augmentation count to 4 and the magnitude to 12. Additionally, we assess the PyTorch implementation of TrivialAugmentWide [37] with default parameters, a straightforward approach that applies a single, random augmentation to each image. Moreover, we introduce 2 proprietary augmentations developed for exploratory purposes. The first, a mild augmentation set, incorporates random affine transformations, perspective adjustments, and rotations. The second, a more intensive augmentation suite, applies random flips, rotations, color jittering, affine transformations, perspective adjustments, and Gaussian blurring, all implemented using the torchvision transformation library. Finally, we evaluate CutMix and MixUp augmentation using the PyTorch v2 implementation, where images are randomly augmented with either CutMix or MixUp using a random selection strategy, ensuring diverse augmentation during training.

### Training Techniques and Postprocessing

Next, we explore various training variations and postprocessing techniques used in this study. Initially, we incorporate multimodal data in our training, which includes both patient images and tabular data detailing wound location, mobility, perception ability, and urinary plus fecal continence. Each factor of mobility, perception, and continence is quantified on a scale from 0 to 4. A joint fusion approach is adopted for multimodal classification, where image embeddings are combined with tabular data embeddings, followed by a final classification head.

Concerning sampling strategies, we address the low sample size in certain classes by employing oversampling techniques to balance class distributions. In addition to classic oversampling, we introduce a synthetic data generation approach by fine-tuning a stable diffusion model to generate artificial images for the minority classes. This allows us to augment underrepresented categories with high-quality synthetic samples. We compare the performance of this approach against traditional oversampling methods to assess its effectiveness in mitigating class imbalance. In terms of postprocessing, we implement ensembling to enhance model performance and robustness by averaging predictions from all 5 folds. Furthermore, test time augmentation is employed by averaging predictions of the original image with 4 additional variants that have undergone mild augmentations such as random flips, rotations, and slight color jitter.

### Evaluation Metrics

To assess the performance of the various models and training strategies, we employ several key metrics. The evaluation metrics used in this study include F1 score, area under the receiver operating characteristic curve (AUROC), and average precision (AP). All metrics were implemented using the torchmetrics library [38]. These metrics were chosen based on informed estimations and insights from Maier-Hein et al [39] recommendations.

### Ethical Considerations

Ethical approval for this study was granted by the Ethics Committee of the Medical Faculty of the University of Duisburg-Essen on October 4, 2022 (ref number: 22‐10905-BO). The study involved retrospective analysis of de-identified image data, and no direct contact with participants occurred. As such, informed consent was not required. All data were processed in compliance with applicable privacy and data protection regulations. In addition, the overall KIADEKU project is registered with the German Clinical Trials Register (Deutsches Register Klinischer Studien (DRKS)) under the registration number DRKS00029961.

## Results

### Overview

Table 2 presents the performance metrics of our best models across the 3 classification tasks. For the binary classification between PU and IAD, the model achieved an F1-score of 93.23%, an AUROC of 0.9852, and an AP of 0.9813. In the PU category classification, the model obtained an F1-score of 75.43%, an AUROC of 0.9384, and an AP of 0.8616. For the IAD category classification, the F1-score was 53.20%, with an AUROC of 0.8391 and an AP of 0.5927.

When examining the optimal combinations per task (refer to Table 3), it is observed that, from an architectural standpoint, transformer models exhibit a superior performance compared to convolution-based models. An exception to this trend is noted in the IAD Category Classification task, where the ConvNeXtV2 model achieves the highest overall performance.

**Table 2.** Performance of the best models.

Technique	F_1_-score	AUROC^a^	AP^b^
Binary	0.9323	0.9852	0.9813
PU^c^ category	0.7543	0.9384	0.8616
IAD^d^ category	0.5320	0.8391	0.5927

a AUROC: area under the receiver operating characteristic curve.

b AP: average precision.

c PU: pressure ulcer.

d IAD: incontinence-associated dermatitis.

**Table 3.** Best benchmark result overview.

Task	Model	Augmentation	Multimodal technique	Sampling technique	Post processing
Binary	TinyViT	TrivialAugmentWide	Cross-attention	None	Ensemble
PU^a^ category	TinyViT	RandAug Strong	None	Synthetic oversampling	Ensemble
IAD^b^ category	ConvNeXtV2	Heavy	None	Synthetic oversampling	Ensemble

a PU: pressure ulcer.

b IAD: incontinence-associated dermatitis.

Regarding augmentations, lighter augmentations enhance performance in the binary classification task. Conversely, the finer category classification tasks benefit from more intensive augmentations, including a heavy augmentation set and significant variations of RandAugment .

Training techniques also show variability across tasks. Multimodality training proves advantageous for the binary classification, whereas it detracts from performance in fine-grained category classification. The cross-attention-based modality fusion approach shows the best performance for the binary classification task. Tailored sampling strategies yield the most substantial performance enhancements, particularly for the PU and IAD category classification tasks, where significant class imbalances are present. Both classic and synthetic oversampling improve performance in these tasks, with the synthetic approach achieving superior results. However, for the binary classification task, neither method provides a noticeable performance increase compared to the standard training regimen.

In the realm of postprocessing techniques, there is a discernible preference for ensembling, which enhances performance across all evaluated tasks. While test-time augmentation also positively impacts performance most of the time, its effectiveness is not as pronounced as that achieved through ensembling.

Detailed performance metrics for the tasks are provided in the multimedia appendices (MultimediaAppendices 1^3^).

**Figure 5.**
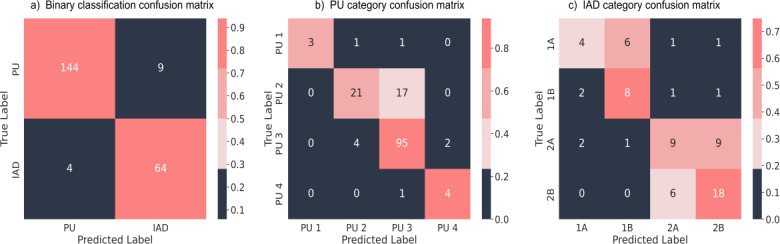
Confusion matrices showing the best benchmarks for different classification tasks: (a) binary classification, (b) pressure ulcer (PU) category, and (c) incontinence-associated dermatitis (IAD) category.

In examining the outcomes of the confusion matrices for the optimal combinations per task, as depicted in Figure 5, a more nuanced understanding of the results and the inherent complexities of the tasks is achieved. The binary classification task demonstrates a high degree of accuracy, achieving low rates of false positives and false negatives, despite the presence of slight class imbalance between the 2 categories. The classification of PU categories presents notable challenges, particularly for categories 1 and 2, which are characterized by their low frequency within the dataset. A mixup between PU-2 and PU-3 is observed to be the most common misclassification, indicating a degree of ambiguity in their differentiation.

A similar pattern is observed in the classification of IAD categories. Categories 1 and 2 prove challenging to classify accurately due to their limited sample sizes. Conversely, categories 3 and 4, while yielding better classification results, also exhibit tendencies for mutual misclassification.

### Performance Comparison

To evaluate our model’s performance, we conducted a comparative analysis against the initial hospital inputs recorded in the primary hospital systems, as well as the annotation performance of 2 wound experts and a health care provider without extensive wound expertise on the test dataset. Since the primary hospital system does not include detailed wound degree information, we limited this comparison to binary classification. Specifically, because the electronic health records in the hospitals only document the presence of PU, we assumed that all other labels correspond to IAD for the purposes of this comparison. Furthermore, we assessed model performance solely on the subset of data labeled as PUs.

As shown in Table 4, our AI model demonstrates a significant improvement in both accuracy and F1 score compared to the initial hospital inputs and health care provider annotations. Notably, the model also slightly outperforms the wound care experts on the test dataset, indicating its potential to assist in clinical decision-making.

In addition to this binary classification analysis, we evaluated the model’s performance on the test datasets with respect to individual wound degree classification, as shown in Table 5. Also, in this more complex classification task, the AI model outperforms the individual wound experts and health care providers.

**Table 4.** Model performance comparison binary.

Method	All images		PU^a^ only
	Accuracy	F_1_-score	Accuracy
AI^b^ model	0.9412	0.9323	0.9532
Primary system	0.8190	0.7260	0.8366
Wound expert 1	0.8959	0.8774	0.9281
Wound expert 2	0.8914	0.8773	0.8889
Health care provider	0.8190	0.7736	0.9150

a PU: pressure ulcer.

b AI: artificial intelligence.

**Table 5.** Model performance comparison for PU^a^ and IAD^b^ categories.

Method	PU category		IAD category	
	Accuracy	F_1_-score	Accuracy	F_1_-score
AI^c^ model	0.8255	0.7543	0.5655	0.5320
Wound expert 1	0.7047	0.5284	0.4328	0.3445
Wound expert 2	0.7181	0.5229	0.3881	0.2941
Health care provider	0.4698	0.3295	0.1642	0.1450

a PU: pressure ulcer.

b IAD: incontinence-associated dermatitis.

c AI: artificial intelligence.

## Discussion

### Principal Findings

In this study, we developed a multimodal deep learning framework for the fine-grained classification of PUs and IAD, along with their respective wound severities. By integrating wound images with categorical patient data, we aimed to enhance diagnostic accuracy and support clinical decision-making in wound care management.

Our extensive evaluations demonstrated that transformer-based architectures, particularly TinyViT, achieved superior performance across the classification tasks. The TinyViT model attained an F_1_-score of 93.23% in the binary classification of PU and IAD, outperforming both wound care experts and nursing staff on the test dataset. This highlights the model’s effectiveness in handling complex visual data and its potential to assist clinicians in accurately distinguishing between these 2 conditions. In the fine-grained classification of PU categories, the TinyViT model again showed the best performance with an F_1_-score of 75.43%. However, the performance was notably lower than in the binary classification task, indicating the increased difficulty in distinguishing between the stages of PU due to subtle visual differences and class imbalances—particularly in differentiating the PU categories stages 1 and 2. Similarly, for IAD category classification, the ConvNeXtV2 model performed best with an F_1_-score of 53.20%, but the overall performance was modest, reflecting challenges in differentiating between IAD severity levels.

These findings indicate that while our models effectively distinguish between PU and IAD, their performance in classifying the specific categories within each condition can be enhanced, particularly due to challenges posed by subtle visual differences and class imbalances. Misclassifications often occurred between adjacent categories, which may be due to overlapping visual features and insufficient samples in certain classes. This underscores the need for larger and more balanced datasets to enhance model training and improve classification accuracy in fine-grained tasks. To address this, future research could focus on targeted data collection to increase underrepresented classes. Additionally, exploring advanced synthetic data generation techniques could provide valuable insights, as our study demonstrated the effectiveness of stable diffusion–based synthetic oversampling.

The integration of multimodal data, which combines images with patient information, was beneficial in the binary classification task, enhancing the model’s ability to differentiate between PU and IAD. This highlights the importance of contextual clinical information in supporting image-based diagnoses. However, the inclusion of multimodal data had less impact on the fine-grained classification tasks. This may be because the categorical patient data do not provide sufficient granularity to assist in distinguishing between wound severities within PU or IAD. Augmentation strategies played a significant role in model performance. Lighter augmentations were more effective for the binary classification task, possibly because they preserved essential image features while providing variability. In contrast, more intensive augmentations benefited the fine-grained classification tasks by helping the models generalize better to subtle variations in wound appearances. This indicates that augmentation techniques should be tailored to the specific requirements of each classification task.

Synthetic data generation and oversampling proved particularly effective in mitigating class imbalances in the PU and IAD category classification tasks, enhancing the model’s ability to learn from underrepresented classes. Notably, the synthetic oversampling approach demonstrated superior performance compared to traditional oversampling, highlighting its potential for improving classification in highly imbalanced settings. In terms of postprocessing, ensembling predictions from multiple folds consistently improved model performance across all tasks, providing more robust and reliable results. While test-time augmentation also contributed to performance gains, its impact was less pronounced compared to ensembling.

These findings contribute valuable insights into the development of more effective diagnostic tools and algorithms for wound classification. By addressing the challenges identified, future work can focus on enhancing the precision and utility of clinical assessments, ultimately improving patient care outcomes.

### Limitations

This study, while providing significant insights into the classification of PU and IAD using advanced AI techniques, has certain limitations that warrant consideration. The dataset used, although comprehensive, may not adequately represent the vast diversity of clinical environments and patient demographics. This could limit the generalizability of the findings to other settings or populations. Additionally, inherent class imbalances within the dataset, despite efforts to mitigate their effects through techniques like oversampling and synthetic data generation, might have influenced the model’s learning, potentially skewing the accuracy toward more frequently represented classes.

Moreover, the integration of multimodal data did not uniformly improve performance, indicating that its effectiveness varies depending on the data’s context and characteristics. This suggests a need for further investigation into which data types are most useful and how they should be integrated.

Furthermore, the study did not exhaustively evaluate every conceivable combination of models, augmentations, training techniques, and postprocessing methods. Instead, selections were based on educated predictions, leveraging the highest-performing techniques from prior phases of the research. This approach, while efficient, may have overlooked potentially effective combinations that could offer further insights or enhanced performance. Additionally, fine-grained classification remains a challenging task due to subtle visual differences between wound categories. Future work should explore attention mechanisms to highlight key image regions and improve model focus, as well as few-shot learning techniques to enhance performance on underrepresented classes. Lastly, the comparison of the model’s performance with wound care experts and primary systems is constrained by the specific test dataset used in this study, and as such, the findings may not be fully generalizable to broader and more diverse datasets or clinical scenarios.

### Conclusions

This study has successfully implemented a framework for classifying PUs and IAD using advanced artificial intelligence methodologies. By systematically evaluating various computational strategies, including different model architectures, augmentation techniques, training methods, and postprocessing approaches, this research provides valuable insights into optimizing AI-driven wound classification models and their potential for real-world clinical application.

The exploration revealed that transformer-based models, notably the TinyViT, generally outperform other architectures, highlighting their suitability for complex visual data processing in fine-grained applications. The effectiveness of different augmentation strategies varied with the complexity of the classification task, emphasizing the need for tailored approaches depending on the specific requirements of the data and the classification objectives.

Furthermore, the study highlights the value of multimodal data integration in enhancing classification accuracy in specific contexts, though its effectiveness varies across tasks. In addition, our findings emphasize the importance of addressing class imbalances, where both classic and synthetic oversampling significantly improved performance, particularly in tasks with severe class disparities. Notably, synthetic oversampling demonstrated superior effectiveness, suggesting that generative models can serve as a powerful tool for augmenting underrepresented classes. Finally, the superior performance of ensembling in postprocessing underscores its potential as a robust strategy for improving prediction reliability, particularly in clinical applications.

In conclusion, our work presents a highly effective classification model capable of accurately distinguishing between PU and IAD images. This model can serve as a valuable tool to assist health care providers in making correct diagnoses, thereby enhancing clinical decision-making and improving patient outcomes in wound care management. The application of our model has the potential to streamline the diagnostic process, reduce the burden on medical staff, and ensure that patients receive appropriate and timely treatment. Furthermore, our extensive benchmarking provides a valuable reference and guidance for future research and development in wound image classification, contributing to the advancement of practical applications within the domain.

## Supplementary material

10.2196/67356 Multimedia Appendix 1 Binary Classification Results.

10.2196/67356 Multimedia Appendix 2 PU Classification Results.

10.2196/67356 Multimedia Appendix 3 IAD Classification Results.

## Abbreviations

AI: artificial intelligence
AP: average precision
AUROC: area under the receiver operating characteristic curve
DRKS: Deutsches Register Klinischer Studien (German Clinical Trials Register)
GLOBIAD: Ghent Global IAD Categorization Tool
IAD: incontinence-associated dermatitis
PU: pressure ulcer
